# An Enhanced Recovery Protocol that Facilitates Same-day Discharge for Simple Laparoscopic Appendectomies

**DOI:** 10.1097/pq9.0000000000000243

**Published:** 2019-12-05

**Authors:** Shabana Z. Shafy, Rebecca Miller, Joshsua C. Uffman, Joseph D. Tobias, Mike Fetzer, Andrew B. Nordin, Brian Kenney, Hina Walia, Giorgio Veneziano

**Affiliations:** From the * Department of Anesthesiology and Pain Medicine, Nationwide Children’s Hospital, Columbus, Ohio; † Department of Anesthesiology and Pain Medicine, the Ohio State University College of Medicine, Columbus, Ohio; ‡ Quality Improvement Services, Nationwide Children’s Hospital, Columbus, Ohio; § Department of Surgery, Nationwide Children’s Hospital, Columbus, Ohio.

## Abstract

Acute appendicitis is the most common gastrointestinal condition requiring urgent operation in the pediatric population with laparoscopic appendectomy (LA) being the current surgical technique. We describe the implementation of a standardized protocol to reduce postoperative nausea and vomiting (PONV) and facilitate same-day discharge after LA.

**Methods:**

A multidisciplinary team developed this protocol to facilitate same-day discharge after observing high rates of overnight stay due to PONV among simple appendectomies performed in 2011–2012. The protocol was implemented in November 2014 and underwent a revision in June 2016. Following the implementation of the protocol, we monitored the patients undergoing an LA at Nationwide Children’s Hospital between November 2014 and August 2017.

**Results:**

We identified 691 patients (255 female) who underwent a simple LA at Nationwide Children’s Hospital between November 2014 and August 2017. The patient population had a median age of 11 years (interquartile range: 9, 14). Among these patients, 514 (74%) were discharged on the day of surgery, and 387 (56%) were protocol compliant. The rate of same-day discharge was higher for compliant cases (79%) than noncompliant cases (69%, *P* = 0.003). Multivariable statistical analysis associated compliance with an increased likelihood of same-day discharge (Odds ratio [OR] = 1.7, 95% CI: 1.2, 2.4, *P* = 0.002).

**Conclusions:**

Implementation of the LA protocol to reduce PONV demonstrated a significant increase in the rate of same-day discharge from the hospital among compliant patients. Also, the adoption of a protocol to select patients for early discharge after LA has shown results with a 45% reduction in the need for inhospital beds.

## INTRODUCTION

Acute appendicitis is the most common gastrointestinal condition requiring urgent surgical intervention in the pediatric population.^1^ Laparoscopic appendectomy (LA) has been shown to decrease morbidity and length of hospitalization when compared with open procedures.^2,3^ As health-care expenditures increase, the emphasis has been placed on decreasing costs while improving the quality and efficiency of clinical care.^4–6^ For appendectomy, minimizing the length of hospital stay has been a key quality metric. Providing LA with hospital discharge on the same day may be challenging as the data show an average postoperative length of stay of 1–2 days.^7^ To improve patient health outcomes, and avoid costs associated with process failures and poor outcomes, surgeons have targeted quality improvement (QI) projects toward increasing the rate of same-day discharge. Previous reports have demonstrated the safety of a same-day discharge protocol for acute appendicitis in children, and high patient satisfaction with this protocol.^8–10^

From 2011 to 2012, we observed high rates of overnight hospitalization (80%) for patients undergoing simple appendectomies. We excluded patients whose appendices were ruptured, gangrenous, or perforated, or if suppuration was noted, or if nonoperative (antibiotic) therapy was attempted before surgery. Among the cases with an overnight stay, we attributed approximately 40% of the hospitalizations to postoperative nausea and vomiting (PONV).

In October 2014, we implemented a standardized protocol for simple laparoscopic appendectomies to facilitate same-day discharge. The protocol standardized perioperative care with interventions designed to minimize the risk of PONV. Evidence-based interventions included intravenous fluid loading, a multimodal medication approach to the prophylactic prevention of PONV, and an opioid-sparing anesthetic. We report the impact of this protocol on same-day discharge rates over a nearly 3-year period (2 years and 10 months) and evaluate the association between protocol compliance and the need for overnight admission. Our secondary aim was to describe the reasons for failure to achieve same-day discharge after protocol implementation.

## METHODS

This study was considered QI work and not human subjects research. Therefore, per policy, the project did not require review and approval by the Institutional Review Board at Nationwide Children’s Hospital (NCH). NCH is a free-standing quaternary-care pediatric hospital performing more than 500 appendectomies each year. The standardized protocol for these procedures (Table 1) was developed by a multidisciplinary team, which included select surgery and anesthesiology faculty, and a manager of QI. After its implementation in November 2014, application of the protocol and compliance was aided by introduction at specialty-specific staff meetings, protocol placards placed in pertinent operating rooms, and outcome reports presented at departmental grand rounds. To further increase compliance, we undertook additional interventions. These interventions included the addition in January 2017 of a clinical decision support reminder in the electronic anesthesia record at the start of any LA and regular individual compliance reports delivered by automated email.

**Table 1.**
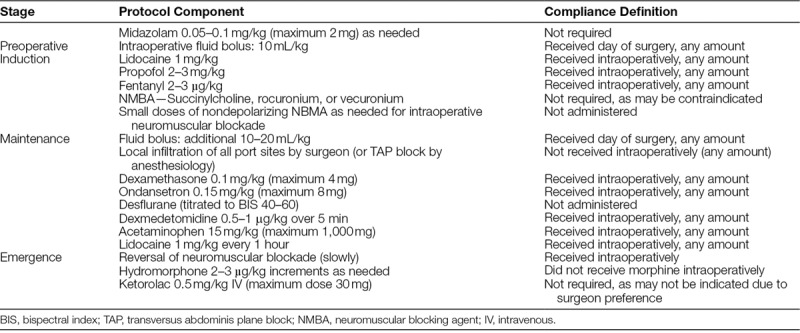
Anesthesia Protocol for LA

Furthermore, we generated emails sent to the anesthesia providers asking them to complete feedback surveys, information on compliance or noncompliance of the protocol, and stating a description of reasons for noncompliance (Table 3). The protocol underwent a revision in June 2016, when we replaced the use of neostigmine in the protocol with sugammadex to reflect a change in our department’s practice of reversal of neuromuscular blockade. In January 2017, we revised the protocol again to remove the desflurane requirement for anesthesia maintenance due to a lack of evidence-based support.

The project team monitored patients undergoing an LA at NCH between November 2014 and August 2017. The QI coordinator in the Department Of Surgery selected simple laparoscopic appendectomies for further review and tracked the intraoperative medication administration and postoperative hospital stay data monthly. We excluded patients with ruptured, gangrenous, perforated, and suppurated appendicitis. The primary outcome was same-day discharge following a simple LA, and the primary aim was to examine protocol compliance. We defined compliance to the protocol as the intraoperative receipt of lidocaine, propofol, dexamethasone, ondansetron, dexmedetomidine, and acetaminophen, no intraoperative receipt of morphine and receipt of intravenous fluids at any point before procedure end. We excluded patients entering the operating room after 5 pm due to the impracticality of discharging patients before midnight on the day of surgery.

Covariates included age, sex, and weight at the time of the procedure. In the descriptive analysis, we summarized categorical data as counts with percentages and continuous data as medians with interquartile ranges (IQRs), according to same-day discharge. Data were compared according to protocol compliance using rank-sum tests for continuous measures and Chi-square tests for categorical measures. In multivariable analysis, we evaluated the association between same-day discharge and compliance with the protocol using logistic regression, adjusted for the covariates listed earlier. In the secondary analysis, we described the reasons for failure to achieve same-day discharge among patients hospitalized overnight. We classified reasons for overnight hospitalization as PONV; inadequate postoperative oral intake; uncontrolled pain; fever; cases with patients presenting with complex syndromes and fibrinogen deficiency; and parental discomfort with early discharge and request for overnight stay at the hospital; and other surgical complications such as retroperitoneal bleed, wound contamination, suppurative appendicitis, gangrenous appendicitis, among others. The proportion of cases that failed to achieve same-day discharge due to PONV was compared according to compliance with the protocol using a test of proportions. We performed statistical analysis using Stata/IC 14.2 (College Station, TX: StataCorp, LP) with 2-tailed *P* < 0.05 considered statistically significant.

## RESULTS

**Fig. 1.**
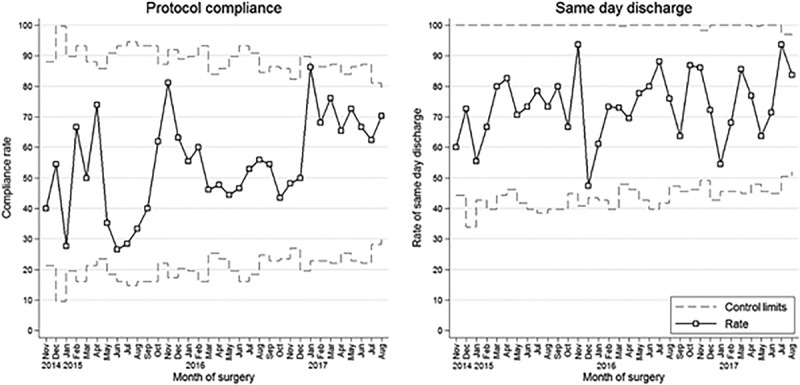
Graph showing the rate of protocol compliance and same-day discharge for each month during the study period, among simple laparoscopic appendectomies performed. All plots show a rate with control limits (solid and dashed lines). A, Protocol compliance. B, The percentage of same-day discharge. Peaks in compliance, such as in November 2015 and January 2017, occurred after interventions to enhance compliance to the protocol, such as displaying the protocol in the operating room, flagging the protocol as a best practice alert on the electronic medical record system, and regularly sending baseline data to the pediatric anesthesiology faculty.

We identified 691 patients (255 female) who underwent a simple LA at NCH between November 2014 and August 2017. The patient population had a median age of 11 years (IQR: 9, 14). Among these patients, 514 (74%) were discharged on the same day, and 387 (56%) were deemed compliant to the protocol. The median length of stay and postoperative length of stay were both 2 days (IQR: 1, 2). Trends of overnight stay and compliance with protocol during the evaluation period are shown in Figure 1. Although the rates of compliance fluctuated over time, the general trend was toward increasing compliance. The percentage of same-day discharge increased over the 4-year study period from 65% in 2014 and 72% in 2015 to 76% in 2016 and 2017. For the majority of months, the percentage of same-day discharge was greater than 60%, with rates peaking at nearly 94%.

**Fig. 2.**
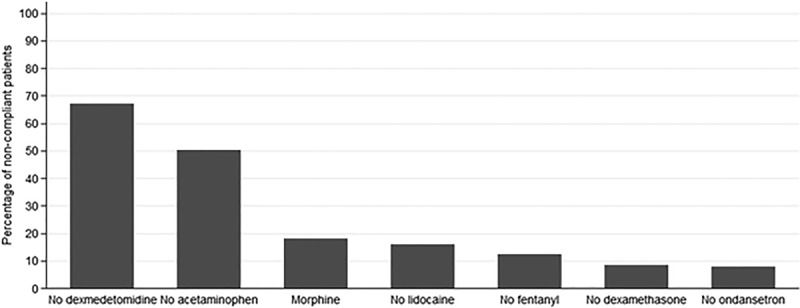
Bar graph showing the most common reasons for noncompliance with the same-day discharge LA protocol with N = 304. The noncompliance was all related to the omission of a medication or the use of morphine instead of hydromorphone or fentanyl.

The most common reasons for noncompliance to the protocol are shown in Figure 2. Among the 304 noncompliant cases, the most common reasons for noncompliance were that patients did not receive dexmedetomidine (67%), did not receive acetaminophen (50%), or received morphine (18%). We compare patient characteristics and outcomes according to protocol compliance in Table 2. The rate of same-day discharge was higher for compliant cases (79%) than noncompliant cases (69%, *P* = 0.003), and patient characteristics did not differ according to case compliance. Multivariable analysis associated compliance with an increased likelihood of same-day discharge (OR = 1.7, 95% CI: 1.2, 2.4, *P* = 0.002). Patient characteristics were not associated with the likelihood of same-day discharge.

**Table 2.**
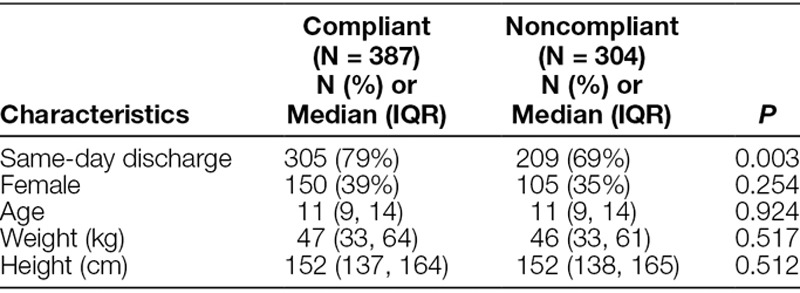
Patient Characteristics and Outcomes, According to Compliance to Protocol (N = 691)

Among the 177 cases that resulted in overnight hospitalization, 83 (47%) had a known reason for hospitalization. Among these cases, 27 (33%) reported uncontrolled pain, 22 (27%) reported PONV, 17 (20%) reported parental discomfort, 4 (5%) reported inadequate postoperative oral intake, 2 (2%) reported fever, 3 (4%) reported patients presenting with complex syndromes; and 13 (16%) reported other surgical complications. None of the patients who were discharged home the same day or surgery required readmission to the hospital for issues related to perioperative care. The 30-day readmission rate was approximately 1% before the implementation of the protocol. The readmission rate subsequently has remained unchanged, demonstrating no increase in returns to the system with the protocol.

## DISCUSSION

Appendicitis is the most common intra-abdominal pathological process in the pediatric population.^1^ Two approaches can be employed to remove the appendix: open appendectomy and LA.^11^ In 1992, Ure et al described a prospective study of 43 patients and concluded that the laparoscopic approach was a safe procedure in children.^12^ Reported advantages of LA have included better exposure of the abdominal cavity, shorter hospitalization, and earlier return to normal activities.^13^ Despite its advantages, LA has posed a challenge to outpatient management, with an average postoperative length of stay of 1–2 days.^7^ However, some studies in pediatric patients with acute appendicitis undergoing appendectomy have demonstrated that same-day discharge is not associated with an increase in 30-day hospital readmission rates or wound complications when compared with discharge 1 or 2 days after surgery.^14^

Enhanced recovery after surgery (ERAS) protocols are multimodal perioperative care pathways designed to decrease complications and achieve early recovery of function after surgical procedures. We obtain this result by maintaining preoperative organ function and reducing trauma and the stress response following surgery. The key elements of ERAS protocols include preadmission counseling and education, optimization of nutrition, standardized analgesic, and anesthetic regimens, and early mobilization. These efforts may improve the recovery process, decrease adverse effects related to anesthetic care, improve analgesia, and decrease hospital length of stay.^15,16^ Implementation of ERAS protocols has been shown to lead to a reduction in complications and hospital stay, improvements in cardiopulmonary function, and earlier resumption of normal activities.^17^

Recent studies have shown promising results for performing nonperforated laparoscopic appendectomies as a “fast-track” or short stay procedure, with a postoperative stay of fewer than 24 hours and the potential for same-day discharge.^18^ Alkhoury et al demonstrated that LA performed on the same day with a mean postoperative stay of 4.5 hours improved patient outcomes and satisfaction without an increase in complication rate or the number of return visits to the emergency department. In general, outpatient surgery may limit the need for hospital resources, decrease cost, and decrease the risk of nosocomial infections, along with the benefit of greater parent satisfaction as a result of early discharge. However, this practice requires a significant change in attitude across multiple layers of hospital organization, including the surgical team, anesthesia providers, pediatricians, and the inpatient nursing staff, to assist in the expeditious discharge of these patients.^9,10^

PONV continues to be one of the leading postoperative complaints from patients. It may not only delay hospital discharge but is the leading cause of readmission to the hospital.^19^ In our institution, we observed high rates of an overnight stay (80%) among simple appendectomies performed in 2011–2012. Of these, we attribute approximately 40% to PONV. The major risk factors for PONV in adults include female sex, nonsmoker status, history of PONV or motion sickness, intraoperative use of volatile anesthetic agents or nitrous oxide, and the administration of opioids.^18^ Established strategies that have demonstrated a reduction in PONV are prophylactic use of antiemetic medications such as ondansetron and dexamethasone; avoidance of general anesthesia by the use of regional anesthesia; avoidance of nitrous oxide; use of propofol; minimization of perioperative opioids; and adequate perioperative hydration.^20–25^ Our multimodal protocol consisted of preoperative anxiolytics (midazolam) as needed, prophylactic antiemetics (ondansetron and dexamethasone), induction with propofol, fluid loading preoperatively and intraoperatively, local anesthetic infiltration at port sites for analgesia, limitation of opioid administration, dexmedetomidine and ketorolac, and the avoidance of nitrous oxide.

In the liberal administration of intravenous fluids, the protocol notably diverged from typical ERAS protocols implemented for elective colorectal surgery. Conservative intravenous fluid management has been a hallmark of colorectal ERAS protocols to decrease bowel edema and encourage the early return of bowel function.^26^ Unlike elective colorectal surgery, the preoperative course for a surgical appendectomy is often marked by nausea, vomiting, and prolonged fasting. These patients may arrive for surgery significantly hypovolemic and in need of intravenous volume restoration. Restoring these patients to euvolemia may improve hemodynamic stability and lead to decreased rates of PONV.^27^

Another unique aspect of the protocol is the use of intravenous lidocaine. Intravenous lidocaine infusions are an established treatment of acute pain in adults. Benefits of intraoperative lidocaine include decreased postoperative pain, earlier return of bowel function, and reduction in PONV.^28^ Recently, several reports demonstrated the safety and efficacy of this analgesic modality in children.^29,30^ In our protocol, given the limited duration of surgery and to conserve resources, we administered lidocaine in a 1-mg/kg bolus every hour, rather than a continuous infusion. The total dose given per protocol was consistent with total doses previously described during continuous infusions in children.

Implementation of the protocol resulted in a significant increase in the rate of same-day discharge from the hospital from 69% to 79% (*P* = 0.003) among patients who were compliant with all elements of the protocol and undergoing an LA. We ascribed the most common reason for noncompliance to nonusage of dexmedetomidine, due to concerns of excessive drowsiness in patients. To address these concerns, we reduced the dose of dexmedetomidine from 1 to 0.5 μ/kg. However, this action did not enhance compliance. Other less common reasons for noncompliance to the protocol included nonusage of acetaminophen, lidocaine, fentanyl, dexamethasone, and ondansetron or the administration of morphine intraoperatively.

Some of the interventions we employed to enhance compliance with our protocol are listed in Table 3. Peaks in compliance, such as those shown in November 2015 and January 2017 in Figure 1, occurred after such interventions. We revised the protocol in June 2016, when the use of neostigmine in the protocol was replaced with sugammadex to reflect a change in our department’s practice of neuromuscular blockade reversal.^31^

**Table 3.**
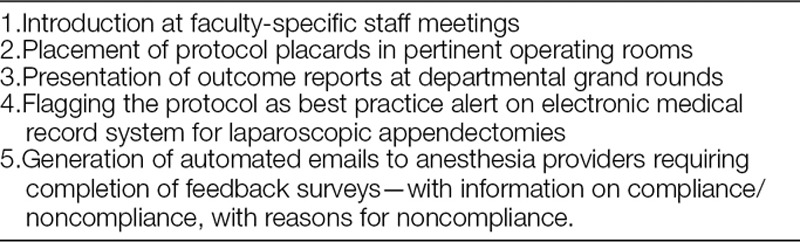
Interventions Implemented to Enhance Protocol Compliance

## CONLCUSIONS

Our analysis supports previously reported studies that LA can be performed as a same-day surgery case or short-stay procedure with numerous potential patient, family, and hospital benefits.^9,10^ Developing a protocol (Table 1) directed toward reducing PONV, one of the leading causes of overnight stay after LA, could result in enhanced rates of same-day discharge. Our data support previous studies that demonstrate that these protocols can be initiated without an increase in perioperative adverse events or return visits to the emergency room. Although earlier hospital discharge can be expected to result in cost savings, the true economic evaluation of such processes must also consider whether the changes in the perioperative care increases cost. For intraoperative care, many of these costs would increase medications such as dexmedetomidine and intravenous acetaminophen, which would be expected to facilitate same-day discharge, although increase medication costs.

## ACKNOWLEDGMENT

We gratefully acknowledge and thank Ronald Sohner and Thomas Chovanec for their participation and contribution to this study.

## DISCLOSURE

The authors have no financial interest to declare in relation to the content of this article.
